# The Human Urinary Proteome Fingerprint Database UPdb

**DOI:** 10.1155/2013/760208

**Published:** 2013-10-09

**Authors:** Holger Husi, Janice B. Barr, Richard J. E. Skipworth, Nathan A. Stephens, Carolyn A. Greig, Henning Wackerhage, Rona Barron, Kenneth C. H. Fearon, James A. Ross

**Affiliations:** ^1^BHF Glasgow Cardiovascular Research Centre, University of Glasgow, 126 University Place, Joseph Black Building, Room B2-21, Glasgow G12 8TA, UK; ^2^Tissue Injury and Repair Group, School of Clinical Sciences and Community Health, University of Edinburgh, 1st Floor Chancellors Building, 49 Little France Crescent, Edinburgh EH16 4SB, UK; ^3^The Roslin Institute & R(D)VS, University of Edinburgh, Edinburgh EH25 9RG, UK; ^4^School of Medical Sciences, University of Aberdeen, Aberdeen AB25 2ZD, UK

## Abstract

The use of human urine as a diagnostic tool has many advantages, such as ease of sample acquisition and noninvasiveness. However, the discovery of novel biomarkers, as well as biomarker patterns, in urine is hindered mainly by a lack of comparable datasets. To fill this gap, we assembled a new urinary fingerprint database. Here, we report the establishment of a human urinary proteomic fingerprint database using urine from 200 individuals analysed by SELDI-TOF (surface enhanced laser desorption ionisation-time of flight) mass spectrometry (MS) on several chip surfaces (SEND, HP50, NP20, Q10, CM10, and IMAC30). The database currently lists 2490 unique peaks/ion species from 1172 nonredundant SELDI analyses in the mass range of 1500 to 150000. All unprocessed mass spectrometric scans are available as “.xml” data files. Additionally, 1384 peaks were included from external studies using CE (capillary electrophoresis)-MS, MALDI (matrix assisted laser desorption/ionisation), and CE-MALDI hybrids. We propose to use this platform as a global resource to share and exchange primary data derived from MS analyses in urinary research.

## 1. Introduction

Screening of human tissues and biofluids for disease biomarkers is an important task in healthcare and disease prevention but is often hindered by the complexity of the system studied, for example, plasma. A substantially less complex system such as urine, which contains approximately 3000 proteins [1, 2], would be a preferred medium to screen for protein or peptide biomarkers as sampling is both simple and noninvasive, and unrestricted quantities are obtainable. Urine is relatively stable in terms of protein/peptide composition and fragmentation state compared with other body fluids such as serum, where proteolytic degradation by endogenous proteases has been shown to occur during or after sample collection [3]. Several investigations have been published describing the urinary peptidome and proteome [4, 5], including biomarker discoveries for several disease processes [6–10]. These studies have used methodologies ranging from traditional 2D gel electrophoresis alone [11] or coupled with mass spectrometry (2-DE-MS) [12], immunohistochemistry [13], liquid chromatography mass spectrometry (LC-MS) [14], and surface enhanced laser desorption ionisation-time of flight mass spectrometry (SELDI-TOF-MS) [15–17].

In complex disease processes, the identification of biomarkers is key to developing novel therapeutic target molecules. Identification of the most robust urinary biomarkers will be enhanced by collating and correlating data from other published and current studies. Currently there are a number of urinary databases available. The majority consists of lists of identified proteins derived from tryptic digests analysed by LC-MS/MS, such as MAPU [18] and Sys-BodyFluid [19] and does not cover naturally occurring mass-centric molecular entities. More recently, a urinary database, combining chromatographic reverse-phase retention times and *m/z* values, has been established [20]. The Mosaiques database [21, 22] consists of naturally occurring protein and peptide patterns detected by capillary electrophoresis MS (CE-MS) from more than 3600 individuals, covering mainly an *m/z* range of 800 to 3000. However, databases that give access to unprocessed data files are not available but would be the most useful resource with which to compare and validate novel datasets.

It is also prudent, especially in urinary proteome research, to remember that any peak in any MS scan profile might be derived from the same molecule (differing only in either its fragmentation or posttranslational modifications). This differentiation might be lost in an MS/MS screen, where proteolytic processing of the samples might alter the original protein/peptide signatures and intensities. Additionally, such fragmentation steps are also time consuming and decrease the sensitivity of the analysis. Other technologies such as ESI (electrospray ionization) methods require off-line fractionation and sample clean-up steps, which can be avoided using LC-MS as a platform. However, the limitation of the inline LC step, usually employing a reverse-phase resin as a solid matrix, narrows the general usability of this method. Alternatives which allow a suitable range of inline fractionation steps using various resins is SELDI, and a novel emerging alternative termed material-enhanced laser desorption/ionization (MELDI) [23, 24], where biomolecules are absorbed onto a solid phase resin and directly used for mass analysis using MALDI. 

We chose the high-throughput SELDI-TOF-MS technology as our platform for biomarker pattern screening. The main advantages of the SELDI technology are its ease of use including little or no sample preparation, high reproducibility, high volume throughput in a minimum of time, with proven methodology over time for the numerous diseases studied, whereas MELDI might require further development before it can be generally applied. The main limitations of the SELDI technology lie with the instrumentation where poor resolution on older instruments led to difficult reproducibility and sometimes questionable results. However, we have chosen a more modern technology (see Section 2). A number of reviews list the issues and compares the various MS-based methods in urinary research [25–27].

Utilizing data from both our own and published studies, we have established the urinary proteome fingerprint database UPdb, which will be publically available as a repository for SELDI-MS data and as a reference for scientists to probe the urinary proteome for proteins implicated in disease processes.

## 2. Materials and Methods

### 2.1. Urine Samples

Urine samples were obtained from 86 cancer patients, 93 noncancer controls, and 21 patients with a previous history of cancer but were diagnosed as cancer-free 6 to 18 months after resectional surgery. Summary participant demographics are shown in Table 1, and full details are provided as part of the database. The cancer sample urines were collected just prior to surgery. One-third of the cancer patients were diagnosed with pancreatic tumours, approximately one-third had oesophageal cancer, approximately one-sixth had malignancies of the oesophagogastric junction (OGJ), and approximately one-sixth suffered from gastric cancer. All procedures were approved by the local research ethics committee. Written informed consent was obtained. The study conformed to the standards set by the Declaration of Helsinki. All urine samples were stored at −40°C.

### 2.2. SELDI-TOF MS

0.1 mL human urine was applied directly to preconditioned SELDI ProteinChip arrays (Bio-Rad Laboratories Inc.) (NP20, H50, SEND, Q10, CM10 and IMAC30), as recommended by the manufacturer, in a ProteinChip bioprocessor and incubated with 0.1 mL binding buffer where appropriate. The chip-spots were washed with 0.2 mL binding buffer three times and air-dried, followed by application of emitter matrix (alpha-cyano-4-hydroxycinnamic acid (CHCA) or sinapinic acid (SPA)). The arrays were read twice, one at low laser settings (focused on 100–50,000 Da *m/z*) and one at high laser settings (focused on 1000–200,000 Da *m/z*), on a ProteinChip Enterprise System PCS4000 (BioRad Laboratories Inc.), SELDI-TOF instrument, and spectral data collected over an average of 588 shots per spot using ProteinChip Data Manager software. Files were exported in “.xml” format. All spectra were processed using the expression difference mapping (EDM) wizard in the ProteinChip Data Manager software (BioRad Laboratories Inc.) with a signal-to-noise-ratio cutoff of 5%, 3% valley depth, and a cluster mass window of 0.2% *m/z*.

## 3. Results and Discussion

SELDI-MS analysis of human urine samples has been reported to show little intra- and interchip variation, as well as low intraindividual day-to-day variation [19] and has been established as a key emerging technology to discover new biomarkers for a variety of diseases. We chose to establish a repository for urinary SELDI data to be made available for the scientific community in order to enable an open exchange of research findings and data sharing.

**Figure 1:**
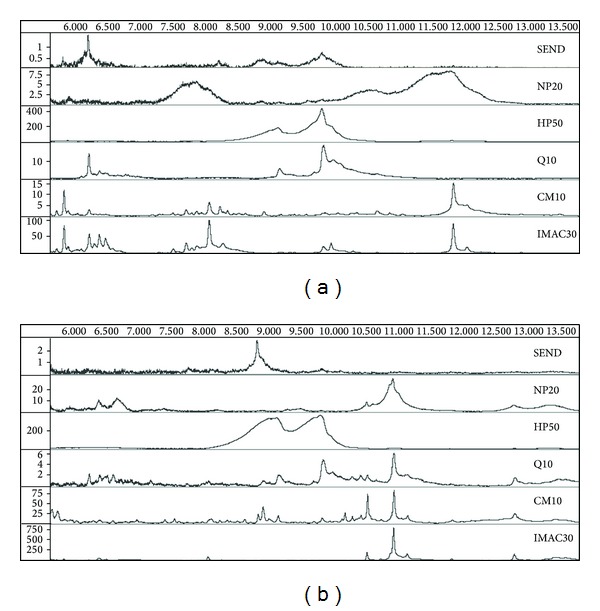
Example of SELDI mass spectra of human urine using various chip surfaces. 0.1 mL urine from a healthy control sample (a) and from a cancer patient (b) was applied to the chip surfaces, as recommended by the manufacturer and analysed by SELDI-TOF. The spectra are plotted as *m/z* (6000 to 13500) against intensity.

We analysed the 200 urine specimens using the SELDI-MS platform on various chip types, ranging from small sized screens of 21 samples on NP20 and HP50 surfaces, medium-sized screens of 63 samples on SEND and Q10 surfaces, and full screens of all 200 samples on CM10 and IMAC30 chip-types (Table 2). The selection of the appropriate chip-surface for a screening purpose depends on many factors, such as peak intensities, distribution, and the number of clearly identifiable ion species (Figure 1). However, under certain conditions a nonoptimal chip type might resolve potential biomarkers and biomarker patterns better than another one. We chose to evaluate all commonly used chip surfaces. 

Both CM10 and IMAC30 (Cu^2+^) gave the best results in terms of signal intensities, peak resolution, and the number of observable peaks. A similar finding has been reported previously using a single urine specimen [16]. Figure 1 shows the SELDI-MS scans of two samples on the six surfaces tested. We also observed that urines from different individuals display a certain degree of heterogeneity, which is easily overcome by increasing the number of analysed samples. Using a 20% threshold for peaks commonly found in any sample, 31.7% of all molecules are present using the IMAC30 (Cu^2+^) chip-type, 25.2% using CM10, and 23.5% using HP50 surfaces. These low numbers are partially due to the various disease states and are higher by comparing samples from healthy control specimens.

**Figure 2:**
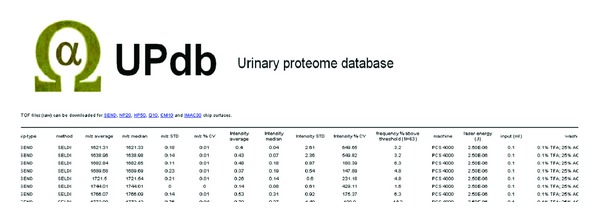
Current content of UPdb. UPdb consists of a list of 3874* m/z* peaks found in human urine in the mass range of 803 to 199000 according to the MS platforms used. The database framework contains data for fractionation methods (separation technique or chip-type, capillary electrophoresis elution time), mass analyzer used (general technique and specific MS instrumentation used), peak specific data (average *m/z* and intensity and frequency above threshold), study-centric information (*N*, number of specimens tested, literature reference linking to PubMed, investigated disease, and species), experimental conditions (input volumes, wash conditions for solid phase extraction methods, and matrix used), protein links (identified protein name, accession number and link to external databases and internal PADB cross-references), and sample specific data (whether the sample was in vitro digested or not, from which tissue it originated, and whether it was further fractionated into subcellular components). Peaks that were classified as biomarkers are indicated in the disease biomarker column, together with a confidence score (*P* value), the regulation (up/down), and fold change in disease as well as substratified frequency %-values in control and disease samples.

Normalising on total ion count and aligning all spectra from individual chip-types resulted in the catalog of 2490 detected peaks, which are fully listed in the database (Figure 2). The database structure also allows the storage and retrieval of information relating to the MS environment, pre- and subfractionation methods, chromatography setups, studied diseases, and other data. Peak-specific data, such as identified biomarker, statistical information, and, if known, identified proteins, are provided. The database covers the mass range of 1500 to 150000 for SELDI spectra and consists of averaged and median *m/z*, intensities and measurement specific data. All 1172 spectra (raw data files) are available for download in “.xml” format from the PADB website at http://www.PADB.org/. 

Initial literature data mining led to the identification of 29 additional urinary datasets, which were incorporated into our database (Table 3). These sets are based on several MS platforms, ranging from SELDI and MALDI to CE-MS and CE-MALDI. The median mass of each individual MS technology, based on the identified peaks per technique, shows that both MALDI and CE-MS favor smaller compounds and peptides, whereas SELDI has an advantage in the higher mass range, albeit with a lower resolution of measured peaks. In total, the database covers a mass range of 800 to 200000 *m/z* or Da since most peaks using these technologies will have a charge of one. Currently, of these 3924 peaks, 39 are associated with identified proteins. This number should continue to rise over time. Additionally, the UPdb database is part of the Proteomic Analysis DataBase (PADB) initiative, and a full integration, as well as development of specific analysis and retrieval tools, is envisaged. 

## 4. Conclusions

UPdb is accessible and downloadable through the PADB initiative at http://www. PADB.org/updb/updb.html. This platform should be used as a global resource to share and exchange primary data derived from SELDI-, MALDI-, MELDI-, CE-, LC-, and other TOF-MS analyses in urinary research. We encourage other laboratories to contribute to UPdb by submitting high quality MS spectra from human urine samples. We envisage providing full linkage of the identified *m/z* species to the large-scale screening resource (LSSR) database (in preparation), which will list molecules identified by MS or other large-scale proteomic methods by their protein or gene names and will also contain a substantial database of identified peptide sequences relating to the proteins listed.

## Figures and Tables

**Table 1:** General overview of the study cohort. 200 patient urine specimens were analysed in this study, derived from 93 healthy individuals (control), 86 cancer patients (cancer), and urine samples from 21 cancer patients 6 months to 1.5 years after surgery (followup). The cancer-types of the 86 cancer patients are listed on the right.

	Control	Cancer	Followup	Total	Oesophagus	OGJ	Pancreas	Gastric	Pancreas/Duodenum	Duodenum	Small bowel
Average age	62	65	68	**64**	66	62	64	74	60	54	71
Male	72	61	12	**145**	22	12	16	9	—	1	1
Female	21	25	9	**55**	5	1	12	6	1	—	—
Total	**93**	**86**	**21**	**200**	**27**	**13**	**28**	**15**	**1**	**1**	**1**

**Table 2:** Overview of SELDI-TOF spectra available as part of the database. Spectra were recorded using low laser energy desorption (*m/z* range 1500 to 25000) and high laser energy desorption (*m/z* range 20000 to 150000) from 200 nonredundant samples. Peak clusters common in all spectra of one chip-type were analysed using a 5% signal-to-noise cutoff, and numbers of peaks found in at least 10% or 20% of all spectra were counted.

Chip-type	Chip specificity	Number of spectra recorded in the low mass range (1500–25000)	Number of spectra recorded in the high mass range (20000–150000)	Demographic distribution (number of patient samples analysed which are healthy/cancer pre-op/disease-free cancer post-op)	Number of peaks above threshold in all samples	Number of common peaks above threshold in 10% of all samples	Number of common peaks above threshold in 20% of all samples
SEND	Reversed phase	63	63	20/43/0	218	25	14
NP20	None (silicon oxide)	21	21	7/14/0	371	166	70
HP50	Hydrophobicity	21	21	7/14/0	362	168	85
Q10	Anion exchanger	63	63	20/43/0	393	120	62
CM10	Cation exchanger	200	200	93/86/21	559	202	141
IMAC30	Metal binding (Cu^2+^)	200	200	93/86/21	587	280	186

**Table 3:** Current number of entries in UPdb by source. All current entries in the UPdb database were tallied based on the MS technique used. The number of detected urinary peaks together with the covered mass range, the median *m/z*, the disease areas studied, the number of identified proteins, and the number of datasets retrieved from the literature are listed.

MS platform	Number of peaks	Mass range *m/z *	Median *m/z *	Disease area	Number of identified proteins	Number of external studies
SELDI	2704	1500–199000	18330	Lupus nephritis, renal allograft nephropathy, cancer, nephritic syndrome, proteinuria, transplant rejection, systemic lupus erythematosus, diabetes, and radiocontrast exposure	27	16
MALDI	45	1220–114000	3212	Cancer	6	3
CE-MS	1125	803–16000	2057	Diabetes, IgA nephropathy, membranous glomerulonephritis, neonatal ureteropelvic junction obstruction, renal damage, renal disease, transplant rejection, and cancer	6	9
CE-MALDI	50	890–6190	2000	Rejection, sepsis, and transplant rejection	0	1

## Abbreviations

2-DE: 2 Dimensional electrophoresis
CE: Capillary electrophoresis
ESI: Electrospray ionisation
LC: Liquid chromatography
MALDI: Matrix assisted laser desorption/ionisation
MELDI: Material-enhanced laser desorption/ionization
MS: Mass spectroscopy
SELDI: Surface enhanced laser desorption ionisation
TOF: Time of flight.
